# Case Report: Novel *UNC93B1* variant causes rheumatoid arthritis and interstitial pneumonia

**DOI:** 10.3389/fimmu.2025.1671984

**Published:** 2025-10-01

**Authors:** Tingyan He, Junbin Ou, Lijuan Huang, Xinyi Zhou, Linlin Wang, Xiaolin Li, Jun Yang

**Affiliations:** ^1^ Department of Rheumatology and Immunology, Shenzhen Children’s Hospital, Shenzhen, China; ^2^ Department of Pediatric Rheumatology and Immunology, Boai Hospital of Zhongshan, Zhongshan, China

**Keywords:** autoimmune disease, systemic lupus erythematosus, monogenic lupus, *UNC93B1*, arthritis

## Abstract

**Background:**

UNC93B1 is a transmembrane protein essential for regulating toll-like receptors (TLRs). Pathogenic variants in human *UNC93B1* have recently been described in a limited number of patients with childhood systemic lupus erythematosus and chilblain lupus.

**Methods:**

Demographic data, medical history, and physical examination findings were obtained. Whole-exome sequencing and Sanger sequencing were performed. The interferon-stimulated gene (ISG) score was analyzed.

**Results:**

We report four patients with a novel *UNC93B1* c.1007G>A p.R336H variant, including three presenting with juvenile arthritis or rheumatoid arthritis, and one with a predominant phenotype of ITP. In addition to arthritis, these patients presented with interstitial pneumonia as the dominant feature. ISG expression analysis during active disease revealed overexpression of IFN-stimulated cytokine genes and an elevated ISG score in P4. To date, 25 cases with *UNC93B1* pathogenic mutations have been reported, including 13 with childhood-onset systemic lupus erythematosus (SLE) and 12 with cutaneous lupus. Management of these patients has varied based on clinical manifestations.

**Conclusion:**

*UNC93B1-*mutation*-*associated disease should be considered in the context of early-onset autoimmune disease, especially childhood-onset SLE, juvenile arthritis, and rheumatoid arthritis. Pulmonary involvement should also be monitored in these patients.

## Introduction

Toll-like receptors (TLRs) recognize pathogen-derived nucleic acids and initiate signals critical for immune responses to infection. A subset of TLRs, such as TLR7 and TLR9, also recognizes self-RNA and self-DNA, respectively. Enhanced TLR7 signaling contributes to autoimmune diseases such as systemic lupus erythematosus (SLE) (1). UNC93B1 is a transmembrane protein essential for regulating TLRs, particularly TLR7, TLR8, and TLR9. UNC93B1 regulates the trafficking of TLRs from the endoplasmic reticulum to endosomes and is contributes to the lysosomal degradation of TLR7 and TLR8 (1–3). *UNC93B1* pathogenic mutations may impair TLR degradation, leading to receptor accumulation, hyper-responsiveness to self-nucleic acids, and hyperactivation of type I interferon and NF-κB signaling pathways (4). Gain-of-function (GOF) human *UNC93B1* variants have recently been described in a limited number of patients with childhood systemic lupus erythematosus and chilblain lupus (1, 4–8). Juvenile arthritis was observed in only one patient (5). To date, as with other newly identified inborn errors of immunity (IEIs), the phenotype of *UNC93B1-*mutation*-*associated disease is not fully understood. Herein, we describe the clinical manifestations of four patients with a novel *UNC93B1* variant to expand the clinical spectrum of *UNC93B1-*mutation*-*associated disease.

## Methods

### Subjects

Demographic data, medical history, and physical examination findings of the patients were obtained through direct interviews and review of patients’ clinical records in Shenzhen Children’s Hospital and Zhongshan Boai Hospital. Written informed consent was obtained based on the principles of the ethics committee of Shenzhen Children’s Hospital.

### Whole exome sequencing

Genomic DNA was extracted from isolated peripheral blood cells. Whole-exome sequencing and Sanger sequencing were performed by MyGenostics (Beijing, China). The FASTQ files were mapped to the human reference genome (hg19). The functional effects of variants were predicted using PolyPhen-2 and MutationTaster, and amino acid conservation across species was analyzed. The structural effects of variants were predicted using the AlphaFold Protein Structure Database and PyMOL.

### Interferon-stimulated gene expression analysis

ISG expression and ISG score were analyzed according to a previously described protocol (9). Total RNA was extracted from PBMCs using the RNAqueous Kit (Thermo Fisher, US) and cDNA was synthesized using Transcriptor First Strand cDNA Synthesis Kit (Roche, Germany) according to the manufacturer’s instructions. The expression of six ISGs and one housekeeping gene was measured by RT-PCR using TaqMan Fast Advanced Master Mix and Gene Expression Assay (FAM) (Thermo Fisher, US) with the following primers: IFI27 (Hs01086370_m1), IFI44L (Hs00199115_m1), IFIT1 (Hs00356631_g1), ISG15 (Hs00192713_m1), RSAD2 (Hs01057264_m1), SIGLEC1 (Hs00988063_m1), and GAPDH (Hs03929097_g1). The relative expression of each gene was normalized to GAPDH and compared with a control sample (a pooled sample from 10 healthy controls), using the 2^−ΔΔCt^ method. The median relative expression of all six ISGs was used to calculate the IFN score for each sample.

## Results

### Clinical manifestations of four patients

**Figure 1:**
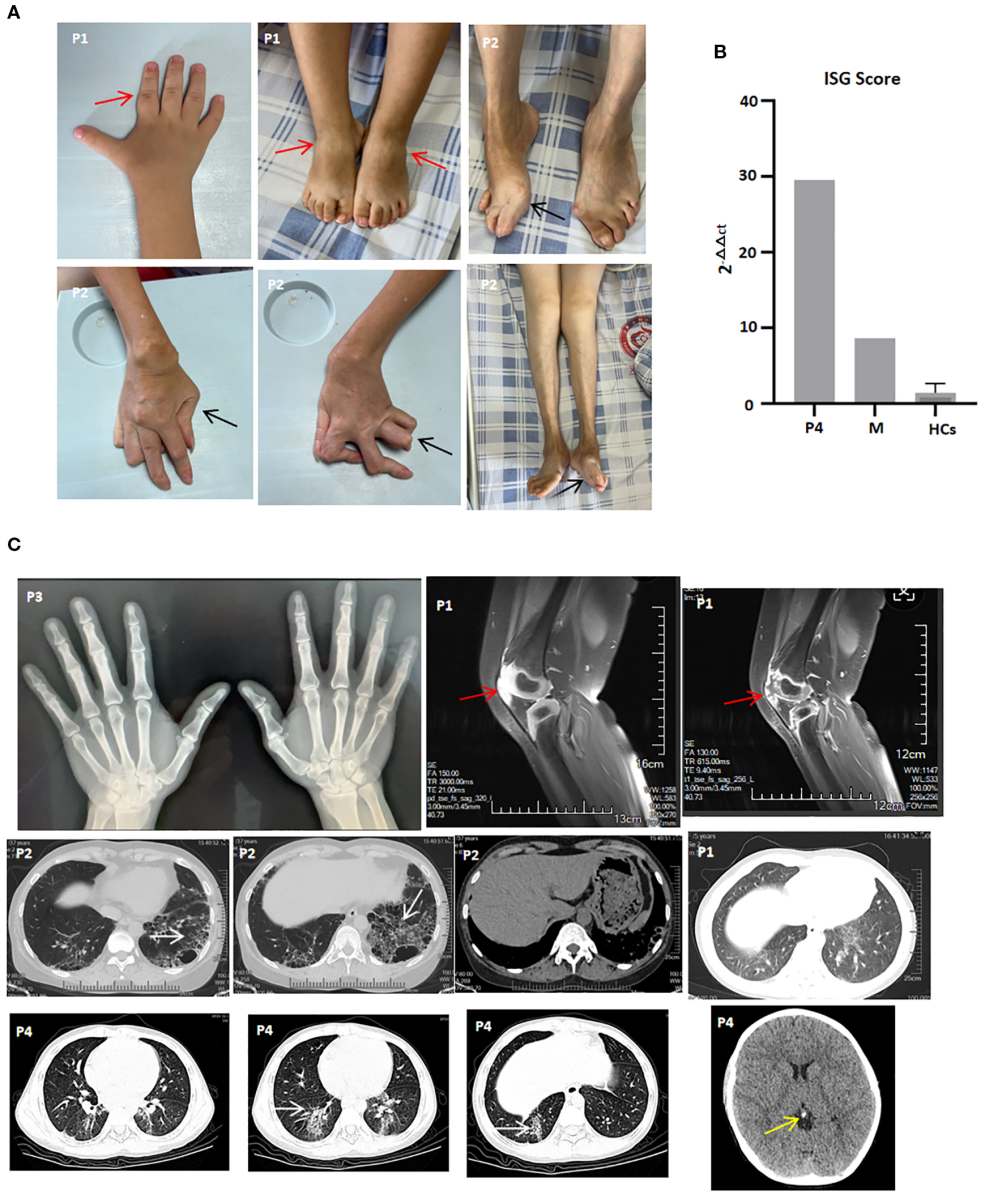
Clinical features and imaging manifestations of patients with the *UNC93B1* c.1007G>A p.R336H heterozygous variation. **(A)** Active arthritis (P1) and joint sequelae (P2). Red arrow indicates joint swelling, and black arrow indicates joint deformity; **(B)** ISG score of P4, his mother (M) and healthy controls (HCs), shown as one representative result of two repeated experiments; **(C)** X-ray of both hands (P3), knee MRI (P1), lung CT (P1, P2, and P4), and brain CT (P4). White arrow indicates interstitial lung diseases, red arrow indicates joint effusions and synovitis, and yellow arrow indicates calcification.

The proband, P1, a six-year-old girl, was born at 34 weeks’ gestational age to non-consanguineous parents. At 14 months of age, developed bilateral knee arthritis, accompanied by touch intolerance and claudication, and gradually developed pain and limited mobility in multiple interphalangeal joints (**Figure 1A**). She was admitted to the Women’s and Children’s Medical Center. Laboratory findings included positive ANA, rheumatoid factor (RF), and anti-CCP antibodies, along with elevated levels of C-reactive protein (CRP; maximum of 48.21 mg/L) and erythrocyte sedimentation rate (ESR; maximum of 43 mm/h). MRI showed effusions and marked synovitis in multiple joints, including the knees, interphalangeal, wrist, ankle, and sacroiliac joints (**Figure 1C**). She was diagnosed with juvenile idiopathic arthritis and treatment with methylprednisolone, methotrexate (MTX), and Enacept, achieving clinical remission for more than 6 months. However, after discontinuing Enacept on her own for three months, she experienced a disease flare and switched to adalimumab, which rapidly resolved the arthralgia. After 13 months of maintenance therapy with adalimumab, she discontinued treatment for financial reasons and redeveloped arthralgia and polyarticular mobility limitation accompanied by interstitial pneumonia. Since then, she has been treated with Enacept and tofacitinib. She had no joint symptoms or signs other than transient wrist and ankle joint pain following respiratory infections.

P2, the proband, was P1’s mother. At two years of age, she began to experience polyarthralgia and subsequently developed polyarticular deformities She had previously received irregular treatment with prednisone, MTX, and nonsteroidal anti-inflammatory drugs (NSAIDs). At 32 years of age, she was referred to Zhongshan Bo Ai Hospital, presenting with ulnar deviation of the hands and feet; swan neck and button deformity of both hands; generalized joint tenderness; and swelling of both knees (**Figure 1A**). Laboratory findings included positive ANA, rheumatoid factor (RF), and anti-CCP antibodies, along with elevated ESR (maximum of 65 mm/h). High-resolution CT of the lungs showed central emphysema in both lobes, multiple bullae, and interstitial fibrosis in the upper and lower lobes of both lungs (**Figure 1C**). During the last two years of follow-up, she did not achieve ACR 30 remission and responded poorly to prednisone, MTX, Enacept, and JAK inhibitors, including tofacitinib, baricitinib, and upadacitinib.

P3, a 40-year-old man, was the proband’s uncle and P2’s older brother. During adolescence, he began to experience arthralgia in the index finger and knee joints. He had previously received prednisone and MTX as initial treatment for three years before self-discontinuing MTX. He was treated with low-dose prednisone and hydroxychloroquine as maintenance therapy. At the last follow-up, although X-rays of both hands showed narrowing of the proximal interphalangeal joint space of the 2nd to 5th fingers, he denied symptoms such as arthralgia, cough, or articular mobility limitation (**Figure 1C**).

P4, a nine-year-old boy, was P3’s son. At the age of seven, he presented with skin purpura and was diagnosed with immune thrombocytopenia (ITP). Laboratory findings included positive ANA and decreased serum C4 levels. CRP and ESR levels were normal. He received intravenous immunoglobulin (IVIG) treatment, after which his thrombocyte levels returned to normal. However, because thrombocytopenia recurred frequently, prednisone and cyclosporin were added as maintenance therapy. Interferon-stimulated gene (ISG) expression analysis during the active disease state revealed overexpression of IFN-stimulated cytokine genes and an elevated IFN score (**Figure 1B**). He switched to mycophenolate mofetil (MMF), tofacitinib, and finally tacrolimus due to dose-dependent prednisone use, poor response to cyclosporin, MMF, and tofacitinib, and the development of interstitial pneumonia (**Figure 1C**). At the last follow-up, he denied symptoms such as arthralgia, cough, or shortness of breath; his thrombocyte levels had returned to normal, and interstitial pneumonia was partially improved.

### *UNC93B1* variation

**Figure 2:**
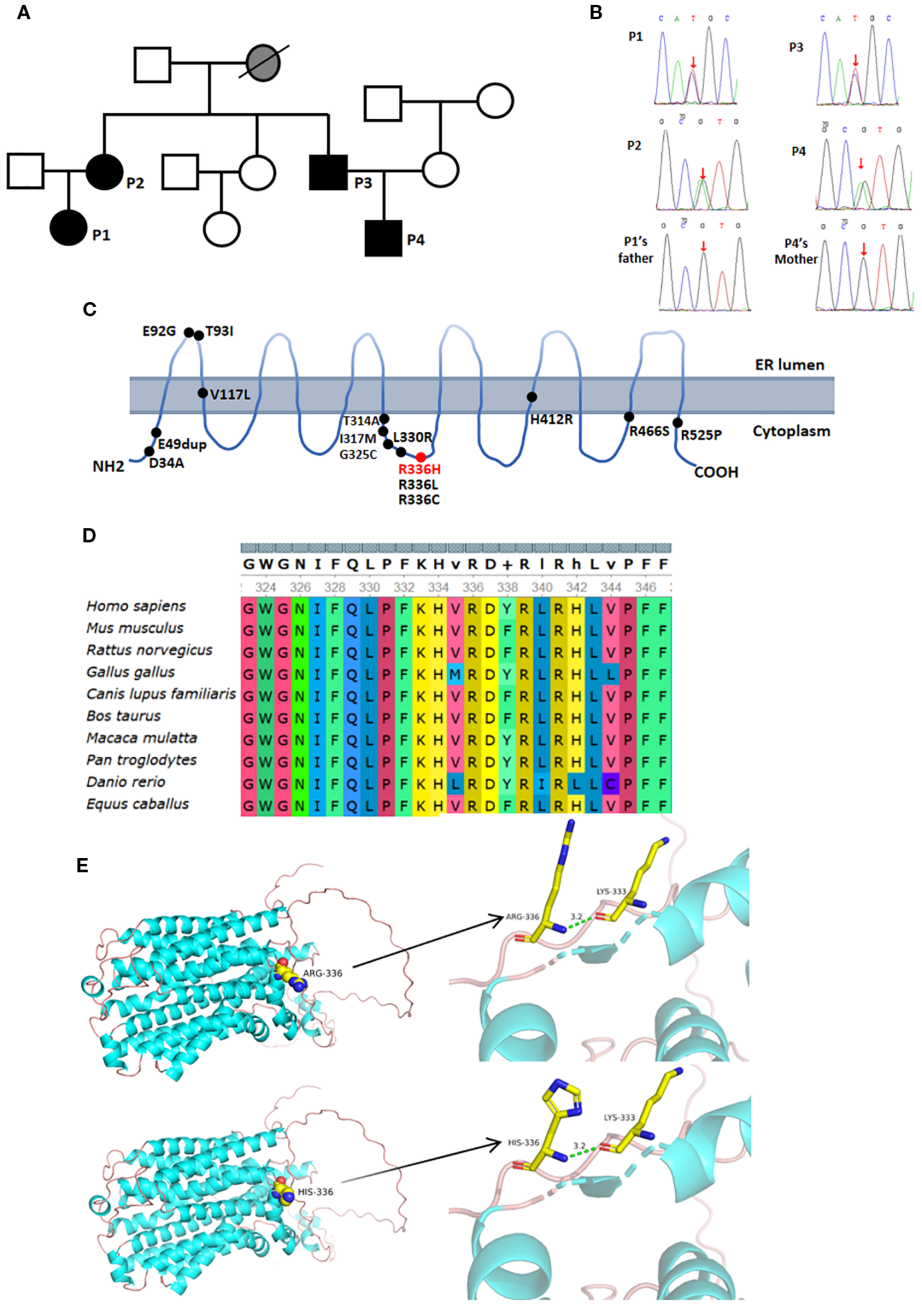
Genetic analysis and imaging manifestations of the patient. **(A)** Family pedigree, black shows patients with the *UNC93B1* c.1007G>A p.R336H heterozygous variation; **(B)** Red arrow indicates the *UNC93B1* c.1007G>A p.R336H heterozygous variation identified by Sanger sequencing; **(C)** Red font denotes the variation in our patients, while black font indicates previously reported variations; **(D)** High conservation of p.R336 across species; **(E)** Protein models of *UNC93B1* with and without the c.1007G>A p.R336H variation.

Whole-exome sequencing revealed a heterozygous *UNC93B1* c.1007G>A p.R336H variant in the four patients, but no variants in healthy family members (**Figures 2A–C**). The predicted values from PolyPhen_2, Mutation Taster, and GERP++ were 0.997, 1, and 5.09, respectively, suggesting probably damaging effects, disease causation, and high conservation (**Figure 2D**). This heterozygous variant was absent from ESP6500si, ExAC_ALL, ExAC_EAS, or Inhouse databases and was further confirmed in all patients by Sanger sequencing. No pathogenic variants were identified in other genes related to autoinflammation or autoimmunity.

The *UNC93B1* c.1007G>A heterozygous variant resulted in an Arg-to-His substitution at amino acid 336, which was predicted by PyMOL not to affect hydrogen bonding (**Figure 2E**).

### Review of literature

To date, 25 cases of *UNC93B1* pathogenic mutation have been reported across five studies, including eight males and 17 females (**Table 1**) (1, 4–7). All patients with available data developed autoimmune disease in childhood. Nearly half of the patients (12 of 25) presented with childhood-onset systemic lupus erythematosus (SLE). Another 12 patients had cutaneous lupus, including one with extended oligoarticular juvenile idiopathic arthritis and a neuroinflammatory movement disorder. One patient presented with autoimmune hemolytic anemia alone. The major immunological abnormalities included positive autoantibodies, decreased C3 and C4 levels, and a positive Coombs’ test. Five patients with cutaneous lupus or familial chilblain lupus were mainly treated with JAK1/2 inhibition. None of the patients received HSCT. Further details are available in **Table 1**.

**Table 1:** An overview of the clinical and immunological findings of patients with *UNC93B1* pathogenic mutation.

No	Sex	Variants	Onset age	Diagnosed age	Dominant disease phenotype	Immunological abnormality	Treatment	Refs.
1	Female	R336H, *Het*	14M	5Y	Juvenile arthritis and interstitial pneumonia	ANA 374 AU/ml (Reference: 0 to 40 AU/ml), anti-dsDNA (-), RF 549.9IU/mL, anti-CCP 201 AU/ml (Reference: 0 to 5 AU/ml), other ENAs(-)	GCs, MTX, Enacept, Tofacitinib, Adalimumab	This study
2	Female	R336H, *Het*	2Y	34Y	Juvenile arthritis → rheumatoid arthritis, polyarticular deformities, interstitial pneumonia,osteoporosis	ANA 1:100, anti-dsDNA (-), RF 193 IU/mL, anti-CCP 62.8 AU/ml (Reference: 0 to 5 AU/ml)	GCs, MTX, Enacept, Tofacitinib→ Baricitinib →Upadacitinib	This study
3	Male	R336H, *Het*	adolescence	40Y	Rheumatoid arthritis, interstitial pneumonia (ND)	NA	GCs, MTX, hydroxychloroquine	This study
4	Male	R336H, *Het*	7Y	9Y	Autoimmune thrombocytopenia and interstitial pneumonia	ANA 1:100, anti-dsDNA (-), other ENAs(-), C4↓	GCs, Tofacitinib, Cyclosporin, MMF, hydroxychloroquine	This study
5	Male	E92G, *Hom*	4M	14Y	childhood-onset SLE	Anticardiolipin-IgG(+), ANA(+), anti-dsDNA (+), C3↓, C4↓	GCs, hydroxychloroquine, cyclosporine A, CTX,MMF, ruxolitinib,	Mishra H et al (1)
6	Female	E92G, *Hom*	2Y	12Y	childhood-onset SLE	Direct Coombs test (+), anticardiolipin-IgG(+), ANA(+), anti-dsDNA (+), anti-SS-A(+), anti-Ro52(+), and anti-U1-snRNP(+), C3↓, C4↓,	GCs, hydroxychloroquine, CTX,MMF, ruxolitinib,	Mishra H et al (1)
7	Male	R336L, *Het*	1.5Y	20Y	ALPS-like, childhood-onset SLE	ANA(+), anti-dsDNA (+), C3↓, C4↓, anti-Cardiolipin-IgM (+), anti-β2-glycoprotein-IgM(+),	GCs, azathioprine, poor medication adherence	Mishra H et al (1)
8	Male	R336L, *Het*	5Y	45Y	ALPS-like, childhood-onset SLE, antiphospholipid antibody syndrome	ANA(+), anti-dsDNA(+), C3↓, C4↓, anti-nucleosome(+), anti-β2-glycoprotein-IgA/G/M(+),	Hydroxychloroquine, azathioprine	Mishra H et al (1)
9	Female	T93I, *Het*	NA	NA	Early onset cutaneous tumid lupus	NA	NA	Rael V E et al (5)
10	Female	T93I, *Het*	NA	NA	Early onset cutaneous tumid lupus	NA	NA	Rael V E et al (5)
11	Male	T93I, *Het*	NA	NA	Early onset cutaneous tumid lupus	NA	NA	Rael V E et al (5)
12	Male	T93I, *Het*	NA	NA	Early onset cutaneous tumid lupus	NA	NA	Rael V E et al (5)
13	Female	R336C, *Het*	3Y	13Y	Extended oligoarticular juvenile idiopathic arthritis, a neuroinflammatory movement disorder, and cutaneous tumid and chillblain lupus rashes	NA	NA	Rael V E et al (5)
14	Female	T314A, *Het*	NA	8Y	childhood-onset SLE	ANA(+), Anti-dsDNA(+), C3↓, C4↓, Anti-Ro/SSA (+),	GCs, CTX,MMF	Al-Azab M et al (7)
15	Female	V117L, *Het*	NA	9Y	childhood-onset SLE	ANA(+), Anti-dsDNA(+), C3↓, C4↓, Anti-Ro/SSA (+), Anti-nRNP(+),	GCs, hydroxychloroquine, CTX, Belimumab	Al-Azab M et al (7)
16	Female	V117L, *Het*	NA	8Y	childhood-onset SLE	ANA(+), Anti-dsDNA(+), C3↓, C4↓, Anti-Ro/SSA (+), Coomb's test(++),	GCs, MMF	Al-Azab M et al (7)
17	Female	V117L, *Het*	NA	10Y	childhood-onset SLE	ANA(+), Anti-dsDNA(+), C3↓, C4↓, Anti-Sm(+), Anti-nRNP(+),	GCs, MTX	Al-Azab M et al (7)
18	Female	V117L, *Het*	NA	10Y	childhood-onset SLE	ANA(+), Anti-dsDNA(+), C3↓, C4↓, Coomb's test(+++),	GCs, hydroxychloroquine, MMF	Al-Azab M et al (7)
19	Female	V117L, *Het*	NA	12Y	childhood-onset SLE	ANA(+), Anti-dsDNA(+), C3↓, Anti-Ro/SSA (+), Coomb's test(+),	GCs, hydroxychloroquine, MMF	Al-Azab M et al (7)
20	Female	V117L, *Het*	NA	13Y	childhood-onset SLE	ANA(+), Anti-dsDNA(+), C3↓, C4↓, Anti-Ro/SSA (+), Coomb's test(++++)	GCs, Cyclosporin	Al-Azab M et al (7)
21	Female	V117L, *Het*	NA	26Y	childhood-onset SLE	C3↓, C4↓, autoantibodies (ND)	NA	Al-Azab M et al (7)
22	Female	I317M, *Hom*	1	NA	Autoimmune hemolytic anaemia, SLE?	ANA(+), anti-dsDNA(+),	NA	David C et al (6)
23	Female	G325C, *Het*	5	NA	Autoimmune hemolytic anaemia,pulmonary artery hypertension, cutaneous lesions on cheeks	ANA(+), anti-RNP(+),	NA	David C et al (6)
24	Male	L330R, *Het*	<1	NA	Chilblain-like lesions on fingers, toes and ears with occasional ulceration	AAb testing negative	NA	David C et al (6)
25	Female	R466S, *Het*	<1	NA	Pruritic/painful cutaneous lesions on hands, feet, mouth ulcers	AAb testing negative	Baricitinib	David C et al (6)
26	Female	R525P, *Het*	6-16	NA	Familial chilblain lupus	NA	JAK1/2 inhibition	David C et al (6)
27	Female	R525P, *Het*	6-16	NA	Familial chilblain lupus	NA	JAK1/2 inhibition	David C et al (6)
28	Male	R525P, *Het*	6-16	NA	Familial chilblain lupus	NA	JAK1/2 inhibition	David C et al (6)
29	Male	R525P, *Het*	6-16	NA	Familial chilblain lupus	NA	JAK1/2 inhibition	David C et al (6)

ND: not detected; NA: not available; ↓: decreased; GCs: Glucocordicoids; MTX: methotrexate; SLE: systemic lupus erythematosus; MMF:mycophenolate mofetil; CTX: Cyclophosphamide.

## Discussion

Herein, we report four patients harboring a novel heterozygous c.1007G>A p.R336H variant in *UNC93B1*, suggesting autosomal dominant inheritance. This variation was absent in healthy family members, is highly conserved, and not found in population databases. At the same amino location, both p.R336L and p.R336C variants in *UNC93B1* have been reported to increase TLR7 and TLR8 responses without affecting TLR9 responses (1, 5, 10). According to the American College of Medical Genetics and Genomics, pathogenicity classification was defined as pathogenic (1 PS + 3 PM + 4 PP) (11). Therefore, the heterozygous p.R336H variant may account for this familial clustering of autoimmune diseases and represents a novel pathogenic variant in *UNC93B1*.

In this study, three patients had Juvenile arthritis or rheumatoid arthritis, except for P4, whose predominant disease phenotype was ITP. JIA has also been reported in a patient with a different p.R336C variant at the same locus. This observation suggests a possible association between pathogenic variants at the p.R336 locus and juvenile arthritis or rheumatoid arthritis. By contrast, pathogenic variants distributed across other domains have been associated with SLE and cutaneous lupus. In summary, identical pathogenic variants may result in varied clinical phenotypes, and different variants may cause the same disease phenotype. Inclusion of additional patients will help clarify potential genotype–phenotype correlations in *UNC93B1-*variant*-*associated disease.

In addition to arthritis, our patients presented with dominant interstitial pneumonia, a feature common in STING-associated vasculopathy with onset in infancy (SAVI) and COPA syndrome (12, 13). Previous studies have shown that UNC93B1 interacts with STING and suppresses STING-activated downstream signaling by delivering STING to the lysosomes for degradation (14, 15). Pathogenic variants at the p.R336 locus may impair STING degradation, leading to overactivation of the cGAS–STING signaling pathway, which could partly contribute to the development of interstitial pneumonia.

*UNC93B1* variant-associated effects include Toll-like receptor-related responses, hyperactivation of the type I interferon response, and overactivation of the NF-κB and cGAS-STING signaling pathways (4). Thus, in addition to autoimmune disease phenotypes such as rheumatoid arthritis and SLE, *UNC93B1* pathogenic variations may theoretically cause autoinflammatory diseases. Inclusion of additional patients in the future will help confirm this possibility.

Proband P1 concurrently received etanercept and tofacitinib for systemic severe inflammation and arthritis. P3 was maintained on low-dose prednisone and hydroxychloroquine. Therefore, management of patients with *UNC93B1-*mutation*-*associated diseases should be individualized according to clinical manifestations. Patients with an SLE phenotype can be treated in line with the ACR SLE guidelines. Patients with chilblain lupus phenotype may receive JAK inhibitors as first-line therapy, which can also be used in cases of interstitial pneumonia, refractory SLE phenotype, or refractory arthritis phenotype. Anifrolimumab, a type I IFN receptor antagonist, may serve as an alternative effective treatment for patients with these phenotypes. Hematopoietic stem cell transplantation has not yet been reported, and gene therapy is not currently available. Further clinical trials with larger patient cohorts will help optimize disease management.

The limitation of this study is the lack of experimental validation for this variation, and the functional link to pathogenicity was defined only by *in silico* predictions and analogy with other variants in *UNC93B1*.

## Conclusion

We have described four patients with a novel pathogenic variant in *UNC93B1*. Their disease phenotypes, including interstitial pneumonia, juvenile arthritis, and rheumatoid arthritis, expand the clinical phenotype spectrum of *UNC93B1-*mutation*-*associated diseases. Management should be individualized.

## Data Availability

The names of the repository/repositories and accession number(s) can be found in the article/supplementary material. Further inquiries about the datasets can be directed to the corresponding authors.
